# Supplementary material for: Is the Concurrent Use of Sorafenib and External Radiotherapy Feasible for Advanced Hepatocellular Carcinoma? A Meta-Analysis
Source: Cancers (Basel). 2021 Jun 10;13(12):2912. doi: 10.3390/cancers13122912 (PMC8230463; doi:10.3390/cancers13122912)
Supplement: Supplementary file 1 [file cancers-13-02912-s001.zip › cancers-1247131-supplementary.pdf]

Supplementary Document 1. Draft of search strategy

Search strategy in Embase and Medline

Embase search:

#1 ('sorafenib'/exp OR sorafenib) AND ('radiotherapy'/exp OR radiotherapy) AND combination AND ('liver cell carcinoma'/exp OR 'liver cell carcinoma')

#2 (cholangio OR 'bile duct'/exp OR 'bile duct' OR (('bile'/exp OR bile) AND duct)) AND ('adjuvant'/exp OR adjuvant OR postoperative) AND ('radiotherapy'/exp OR radiotherapy OR 'radiation'/exp OR radiation OR 'chemoradiotherapy'/exp OR chemoradiotherapy) AND ('Article'/it OR 'Article in Press'/it)

#3 (cholangio OR 'bile duct'/exp OR 'bile duct' OR (('bile'/exp OR bile) AND duct)) AND ('adjuvant'/exp OR adjuvant OR postoperative) AND ('radiotherapy'/exp OR radiotherapy OR 'radiation'/exp OR radiation OR 'chemoradiotherapy'/exp OR chemoradiotherapy) AND ('Article'/it OR 'Article in Press'/it) ('article'/it OR 'article in press'/it) NOT ('animal experiment'/de OR 'animal model'/de OR 'animal tissue'/de OR 'case report'/de OR 'human cell'/de OR 'human tissue'/de OR 'in vitro study'/de OR 'in vivo study'/de OR 'nonhuman'/de)

*#1 is our basic strategy using Emtree; #2 is to filter studies with irrelevant formats (e.g. reviews, editorials, letters, conference abstracts); #3 is to filter studies which is not relevant clinical studies (e.g. case reports, in vivo studies, systematic reives)

Search strategy in Pubmed and Cochrane library

Search term used: sorafenib AND radiotherapy AND combined AND hepatocellular

Using search query, filters to include clinical trial (I~IV), comparative study, clinical study, controlled clinical trial, multicenter study, and observational study were used. We did not use any filter in Cochrane library.

Supplement Table 1. Scoring sheet according to New-Castle Ottawa scale

	Selection				Comparability	Outcome			**Overall score (9 to be full)**
	1	2	3	4	1	1	2	3	
	Representativeness of the exposed cohort	Selection of the non exposed cohort	Ascertainment of exposure	Outcome of interest was not present at start of study	Comparability of cohorts on the basis of the design or analysis	Assessment of outcome	Was follow-up long enough for outcomes to occur	Adequacy of follow up of cohorts	
Sun	1	1	1	1	1	1	1	1	8
Wada	1	1	1	1	2	1	1	1	9
Zhao	1	1	1	1	2	1	1	1	9
Kang	1	1	1	1	2	1	1	1	9
Liu	1	1	1	1	2	1	1	1	9
Zhang	1	1	1	1	2	1	1	1	9
Brade	1	1	1	1	0	1	1	1	7
Cha	1	1	1	1	0	1	1	1	7
ChenB	1	1	1	1	0	1	1	1	7
ChenWS	1	1	1	1	0	1	1	1	7
Li	1	1	1	1	0	1	1	1	7
